# Factors Predicting Difficulty of Laparoscopic Low Anterior Resection for Rectal Cancer with Total Mesorectal Excision and Double Stapling Technique

**DOI:** 10.1371/journal.pone.0151773

**Published:** 2016-03-18

**Authors:** Weiping Chen, Qiken Li, Yongtian Fan, Dechuan Li, Lai Jiang, Pengnian Qiu, Lilong Tang

**Affiliations:** 1 Department of Colorectal Surgery, Zhejiang Cancer Hospital, Hangzhou, Zhejiang, 310022, China; 2 Department of Radiology, Zhejiang Cancer Hospital, Hangzhou, Zhejiang, 310022, China; The Chinese University of Hong Kong, HONG KONG

## Abstract

**Background:**

Laparoscopic sphincter-preserving low anterior resection for rectal cancer is a surgery demanding great skill. Immense efforts have been devoted to identifying factors that can predict operative difficulty, but the results are inconsistent.

**Objective:**

Our study was conducted to screen patients’ factors to build models for predicting the operative difficulty using well controlled data.

**Method:**

We retrospectively reviewed records of 199 consecutive patients who had rectal cancers 5–8 cm from the anal verge. All underwent laparoscopic sphincter-preserving low anterior resections with total mesorectal excision (TME) and double stapling technique (DST). Data of 155 patients from one surgeon were utilized to build models to predict standardized endpoints (operative time, blood loss) and postoperative morbidity. Data of 44 patients from other surgeons were used to test the predictability of the built models.

**Results:**

Our results showed prior abdominal surgery, preoperative chemoradiotherapy, tumor distance to anal verge, interspinous distance, and BMI were predictors for the standardized operative times. Gender and tumor maximum diameter were related to the standardized blood loss. Temporary diversion and tumor diameter were predictors for postoperative morbidity. The model constructed for the operative time demonstrated excellent predictability for patients from different surgeons.

**Conclusions:**

With a well-controlled patient population, we have built a predictable model to estimate operative difficulty. The standardized operative time will make it possible to significantly increase sample size and build more reliable models to predict operative difficulty for clinical use.

## Introduction

Colorectal cancer is one of the most common malignancies and one of leading causes of cancer death in U.S. and worldwide [1, 2]. Surgical resection is a standard treatment for patients with non-metastatic colorectal cancer. Laparoscopic resection for colorectal cancer has similar short [3] and long term [4–6] outcomes as conventional open surgery, but with more clinical advantages [3, 7]. Laparoscopic surgery allows for a shorter time interval when initiating chemotherapy following surgery, which improves colon cancer patient survival [8]. After its initial introduction in 2001, laparoscopic surgery for colorectal cancer has been extensively utilized throughout China [9]. Recent studies suggest that laparoscopic surgery for colorectal cancer has a great potential to be more widely applied within U.S. in the future [10, 11].

Laparoscopic surgery for low rectal cancer requires advanced laparoscopic surgical skills because the operation is performed within the narrow pelvic cavity [12]. A surgeon’s advanced laparoscopic skills are one of the most important factors for operative success. Existence of learning curves suggests that surgeons develop laparoscopic skills through continuous repetition of surgical procedures. In addition, patients’ preoperative clinical, anatomical and pathological factors, such as body mass index (BMI), pelvic size, preoperative chemoradiotherapy and tumor distance to anal verge, have been related to operative difficulties in previous studies [13–18]. Great efforts have been devoted to identify patient’s factors for building models that predict the difficulty of performing laparoscopic low anterior resection for rectal cancer. The predicted operative difficulty by these models are valuable in informing patients of the possible risks and complications that could occur both during and after surgery. Surgical residents could select suitable cases during their training, thus minimizing poor outcomes caused by inexperience. However, these results are still not consistent. Limited sample size and confounding factors such as different surgical procedures and surgeons with variable experiences may be the causes of the inconsistencies.

The purpose of our current study was to screen patients’ clinical, anatomical and pathological factors that contribute to the difficulty of laparoscopic resection in low rectal cancer. To minimize the inclusion of confounding factors, all patients had rectal cancer 5–8 cm from anal verge and underwent laparoscopic sphincter-preserving anterior resections with total mesorectal excision (TME) and double stapling technique (DST). Data of patients, whose surgery was performed by one experienced surgeon, were utilized to build models to predict the operative difficulty. Predictability of the model was then determined by data from other surgeons.

## Material and Methods

### Patient selection

From December 2008 to November 2014, there were a total of 199 consecutive patients who had rectal cancer 5–8 cm of distance from the anal verge, and underwent laparoscopic sphincter-preserving low anterior resection with TME and DST. Among these patients, 155 operations were performed by one surgeon (W.C.), whereas 44 patients were conducted by other surgeons (Q.L., Y.F., and D.L.). The date was selected when all four surgeons had at least two years’ experiences in laparoscopic surgeries and performed over 50 laparoscopic operations on colorectal cancers. All patients with rectal cancers of local perforation, infiltration to adjacent organs, or distant metastasis, were excluded for laparoscopic surgery. This study was approved by the Research and Ethics Committee of Zhejiang Cancer Hospital. Due to the retrospective nature of the study, informed consent was waived by the Committee. Identifying information of all patients including names and hospital numbers were omitted prior to data analysis.

**Fig 1:**
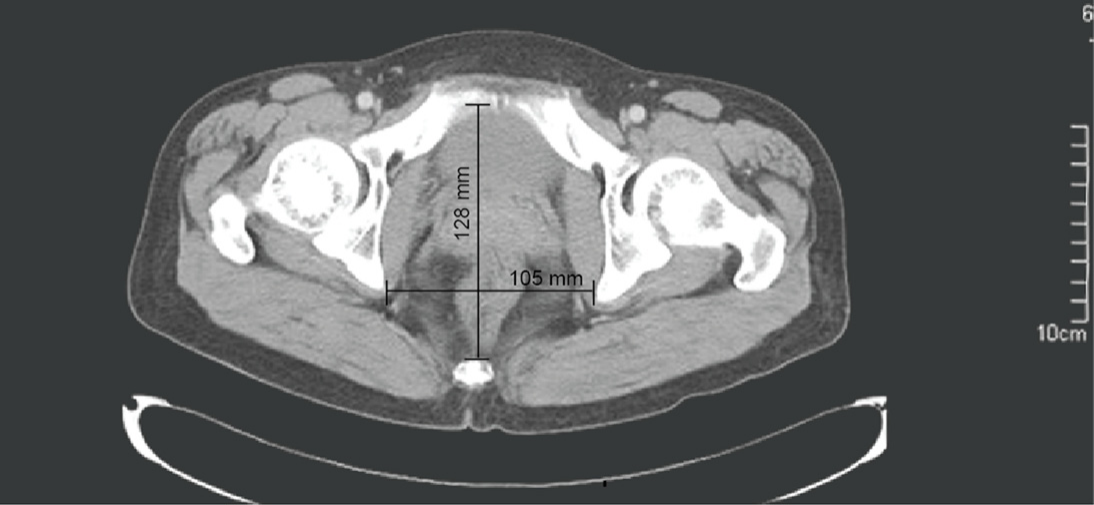
Measurements of pelvic dimensions: axial CT image showed a transverse interspinous distance of 105 mm, and anteroposterior sacrum-pubis distance of 128 mm.

Patient’s age, gender, BMI, prior abdominal surgery, concurrent diseases (hypertension and/or diabetes), preoperative chemoradiotherapy, operative time, amount of blood loss during surgery, morbidity, duration of hospital stay after surgery, number of harvested lymph nodes, and tumor size and staging, were collected from the medical records. Pelvic data, including interspinous distance (the narrowest distance between the ischial spines) and sacrum–pubis distance (the distance from the pubic symphysis to the sacrum at the level of ischial spines) (Fig 1) were blindly measured on axial CT images by a radiologist (L.T.). Postoperative pathological results were used to provide a precise description of the tumors (diameter, degree of circumferential occupation and stage). Tumors were staged based upon the sixth or seventh tumor–node–metastasis (TNM) classification of the Union for International Cancer Control (UICC). Operative time was calculated from the electronic record on the anesthesia machine from the start of creating a pneumoperitoneum to the end when the abdominal incision being sutured. Anastomotic leakage was diagnosed by the presence of symptoms described previously [19].

### Surgical procedure

Laparoscopic surgeries were all performed using similar procedures, as described previously, but with minor modifications [13]. All patients’ hips were raised by 5cm with a pad during surgery. The primary operative hole, with 12 mm trocar, was placed approximately 3 cm below the right lower quadrant of McBurney’s point, to avoid injury to the inferior epigastric and external iliac vessels. Both rectal inflation and anastomotic bleeding were checked before closure of the abdomen.

### Statistical analysis

Quantitative data were presented as mean ± standard deviation. Data transformation by square root was applied for the operative time and blood loss, in order to meet the normality requirement. Operative time and blood loss were standardized by minusing the mean and then dividing the standard deviation. Student’s t-test or Chi Square test was applied to examine the gender difference in variables as indicated.

Using the data of 155 patients whose surgery was performed by the same surgeon (W.C.), we analyzed the relationships between the patients’ variables and endpoints (standardized operative time, blood loss during surgery and morbidity after surgery) through linear or logistic regression models. After univariate analysis, variables with a P value less than 0.25 were selected for multivariate analysis. Multivariate analysis was performed using a stepwise method.

Data gathered from another 44 patients were used to validate the predictability of the built models. The predictability was determined by linear regression analysis based upon the correlation between actual and predicted values. The actual standardized operative time was calculated by minusing the mean and then dividing the standard deviation. The predicted standardized operative time was calculated based on the built model.

The statistical analysis was performed using SAS software (SAS Institute Inc., Cary, NC), and P < 0.05 was considered to be significant.

## Results

### Patients and tumor characteristics

Among the 155 patients, 107 (69.0%) of them were male and 48 (31.0%) were female. The mean age of all patients was 57.9 ± 10.2 years. Male patients were 4.6 years on average older than female patients (P = 0.01). The mean body mass index was 22.4 ± 2.9. There was no significant difference between male and female patients. Both pelvic parameters, interspinous and sacrum–pubis distance, were significantly larger in females, compared to males (P = 0.0001 and P<0.0001, respectively) (Table 1).

**Table 1:** Patients’ demographic and anthropomorphic features, intraoperative and postoperative outcomes.

	Overall	Male	Female	
	155	107 (69.0%)	48 (31.0%)	P
Age (years)	57.9 ± 10.2	59.3 ± 9.0	54.7 ± 12.1	0.01
BMI (kg/m^2^)	22.4 ± 2.9	22.5 ± 2.9	22.1 ± 2.6	0.37
Interspinous distance (mm)	102.5 ± 10.9	99.1 ± 10.2	106.2 ± 11.2	0.0001
Sacrum–Pubis (mm)	101.0 ± 11.8	95.8 ± 8.1	112.6 ± 10.3	<0.0001
Preoperative chemoradiotherapy	16 (10.3%)	14 (13.1%)	2 (4.2%)	0.09
Concurrent diseases	15 (9.7%)	11 (10.3%)	4 (8.3%)	0.70
Prior abdominal surgery	24 (15.5%)	11 (10.3%)	13 (27.1%)	0.002
Operative time (mins)	166.5 ± 62.8	170.4 ± 63.7	157.7 ± 61.0	0.25
Blood loss (ml)	67.2 ± 43.9	60.9 ± 37.5	81.4 ± 53.4	0.007
Postoperative hospital stay (days)	10.7 ± 3.5	11.1 ± 3.9	9.9 ± 2.5	0.06
Temporary diversion	74 (47.7%)	51 (47.6%)	23 (47.9%)	0.98
Morbidity	15 (9.7%)	10 (9.3%)	5 (10.4%)	0.83

Continuous data presented as mean ± standard deviation were analyzed by student t-test, whereas categorical data were examined by Chi-Square test.

Preoperative chemoradiotherapy was only administered in 16 (10.3%) patients (Table 1). Concurrent diseases (hypertension and/or diabetes) occurred in 15 (9.7%) patients. A total of 24 (15.5%) patients had prior abdominal surgery. Females (27.1%) had a significantly higher rate of prior abdominal surgery than males (10.3%) (P = 0.002).

Average operative time was 166.5 ± 62.8 minutes. Operative time in males was 12.7 minutes longer than females, but was not significant (Table 1). Average blood loss during surgery was 67.2 ± 43.9 ml. Males had a blood loss of 60.9 ± 37.5 ml, which was significantly less than that of females (81.4 ± 53.4) (P = 0.007). When patients were divided into 5 groups (31 patients/group) based chronologically on operation dates, we found that operative time and blood loss were not significantly different among groups. Males (11.1 ± 3.9 days) had a longer hospital stay following surgery, than females (9.9 ± 2.5 days) (P = 0.06).

Temporary diversion (a diverting ileostomy) was created in 74 patients. This was based upon the existence of possible anastomotic leaking risks: unsatisfactory anastomosis, patients with preoperative chemoradiotherapy, diabetes, malnutrition, or chronic incomplete intestinal obstruction.

Postoperative morbidity rate was relatively low. Ten male patients (9.3%) and 5 female patients (10.4%) developed morbidity after surgery (Table 1). These morbidities included 3 cases of anastomotic bleeding, 4 cases of anastomotic leakage, one case of intestinal obstruction and anastomotic leak, and 7 cases of infection to the wound and other sites. No positive longitudinal resection margins were identified. No conversion to open surgery happened and no patient died from the surgery.

Pathological information of tumors was summarized in Table 2. Male and female patients had similar features in tumor diameter, portion of circumferential wall, number of lymph nodes collected, staging, and TNM phase. Females had significantly more tumors 6 cm away from anal verge, whereas male had more tumors 8 cm from anal verge (P = 0.01).

**Table 2:** Anatomopathological features of tumors.

	Overall	Male	Female	P
Tumor diameter	5.0 ± 1.8	5.0 ± 1.9	4.9 ± 1.6	0.83
Circumferential occupation	0.6 ± 0.2	0.6 ± 0.2	0.5 ± 0.2	0.06
Harvested lymph nodes	17.3 ± 7.9	17.4 ± 8.4	17.3 ± 6.4	0.97
Stages				
0	9 (5.8%)	7 (6.5%)	2 (4.1%)	
I	40 (25.8%)	27 (25.3%)	13 (27.1%)	
II	33 (21.3%)	23 (21.5%)	10 (20.8%)	
III	73 (47.1%)	50 (46.7%)	23 (47.9%)	0.94
T				
0	14 (9.0%)	10 (9.3%)	4 (8.3%)	
1	12 (7.7%)	8 (7.5%)	4 (8.3%)	
2	32 (20.6%)	21 (19.6%)	11 (22.9%)	
3	91 (58.7%)	65 (60.7%)	26 (54.2%)	
4	5 (3.9%)	3 (2.8%)	3 (6.3%)	0.83
N				
0	83 (53.5%)	59 (55.1%)	24 (50.0%)	
1	44 (28.4%)	32 (29.9%)	12 (25.0%)	
2	28 (18.1%)	16 (15.0%)	12 (25.0%)	0.32
Anal verge (cm)				
5	29 (18.7%)	21 (19.6%)	8 (16.7%)	
6	35 (22.6%)	16 (15.0%)	19 (39.6%)	
7	33 (21.3%)	23 (21.5%)	10 (20.8%)	
8	58 (37.4%)	47 (43.9%)	11 (22.9%)	0.005

Continuous data presented as mean ± standard deviation were analyzed by T-test, whereas categorical data were examined by Chi-Square test.

### Factors related to operative difficulty in overall patients

Univariate analysis showed that prior abdominal surgery (P = 0.01), temporary diversion (P = 0.03), preoperative chemoradiotherapy (P<0.0001), tumor distance from anal verge (P = 0.0001), BMI (P<0.0001), and interspinous distance (P<0.0001) were significantly associated with the standardized operative time. Multivariate analysis showed prior abdominal surgery (estimate = 0.49, P = 0.01), preoperative chemoradiotherapy (estimate = 0.55, P = 0.02), tumor distance to anal verge (estimate = -0.14, P = 0.02), BMI (estimate 0.10, P<0.0001) and interspinous distance (estimate = -0.02, P = 0.0002) were predictors for the standardized operative time (Table 3).

**Table 3:** Determinants for standardized operative time.

Variables		Estimate (SE)	P Values
Univariate			
Prior Abdominal surgery		0.60 (0.22)	0.01
Temporary diversion		0.36 (0.16)	0.03
Preoperative chemoradiotherapy		1.10 (0.24)	<0.0001
Distance to anal verge		-0.27 (0.07)	0.0001
BMI		0.11 (0.03)	<0.0001
Interspinous distance		-0.03 (0.01)	<0.0001
Multivariate			
Intercept		1.14 (0.04)	<0.0001
Prior abdominal surgery		0.49 (0.19)	0.01
Preoperative chemoradiotherapy		0.55 (0.20)	0.02
BMI		0.10 (0.02)	<0.0001
Interspinous distance		-0.02 (0.01)	0.0002
Distance to anal verge		-0.14 (0.06)	0.02

SE: standard error

Based upon this multivariate model, we generated a formula to calculate the standardized operative time: 1.14+ 0.49 X prior abdominal surgery + 0.55 X preoperative chemoradiotherapy + 0.10 X BMI -0.14 X tumor distance from anal verge– 0.02 X interspinous distance (mm). For a patient with prior abdominal surgery, preoperative chemoradiotherapy, BMI of 30, interspinous distance of 90 mm and a rectal cancer 5 cm from anal verge, his/her estimated standardized operative time is 1.14 + 0.49 + 0.55 + 0.1 X 30–0.14 X5–0.02 X 90 = 2.68. Percentile ranking of a value calculated from this formula can then be found from the Z score chart. For the value of 2.68, the percentile ranking is 99.26; In other words, it would be extremely difficult for him to undergo the laparoscopic surgery.

Our results showed that gender (P = 0.02), tumor diameter (P = 0.046) and preoperative chemoradiotherapy (P = 0.02) were the predictors for blood loss by univariate linear regression analysis (Table 4). Gender (estimate = 0.42, P = 0.02) and tumor diameter (estimate = 0.09, P = 0.04) were predictors for blood loss by multivariate analysis (Table 4). This result suggests that blood loss is not an ideal endpoint to identify predictable patients’ factors.

**Table 4:** Determinants for standardized blood loss.

Variables		Estimate (SE)	P values
Univariate			
	Gender	0.41 (0.17)	0.02
Tumor diameter		0.09 (0.04)	0.046
Preoperative chemoradiotherapy		0.36 (0.13)	0.02
Multivariate			
Intercept		-0.99 (0.32)	0.002
Gender*		0.42 (0.17)	0.02
Tumor diameter		0.09 (0.04)	0.04

* Male = 1 and female = 2

SE: standard error

Univariate logistic regression analysis showed that temporary diversion (P = 0.0462), the degree of tumor circumferential occupation (P = 0.02), and tumor diameter (P = 0.02) were predictors for postoperative morbidity. Temporary diversion (estimate = 1.67, P = 0.047) and tumor diameter (estimate = 0.44, P = 0.02) were predictors for postoperative morbidity in a multivariate analysis (Table 5). Due to its relative low rate in our study, morbidity is not a good endpoint to predict the operative difficulty.

**Table 5:** Determinants for postoperative morbidity.

Variables		Estimate (SE)	P values
Univariate			
Temporary diversion		1.21 (0.61)	0.046
Circumferential occupation		0.28 (0.12)	0.02
Tumor diameter		0.35 (0.15)	0.02
Multivariate			
Intercept		-4.93 (1.06)	<0.0001
Temporary diversion		1.67 (0.78)	0.047
Tumor diameter		0.44 (0.18)	0.02

SE: standard error

### Factors influencing the operative difficulty are different in males and females

Based on the univariate analysis, prior abdominal surgery (P = 0.003), preoperative chemoradiotherapy (P = 0.0002), temporary diversion (P = 0.002), tumor distance from anal verge (P = 0.0001), BMI (P<0.0001) and interspinous distance (P = 0.0002) were significantly associated with operative time in males. Prior abdominal surgery (estimate = 0.79, P = 0.003), preoperative chemoradiotherapy (estimate = 0.72, P = 0.004), BMI (estimate = 0.10, P = 0.0003) and interspinous distance (estimate = -0.02, P = 0.001) were significantly associated with the standardized operative time in males, based on multivariate analysis (Table 6). In females, interspinous distance (P = 0.04) was associated with the standardized operative time, whereas age (P = 0.05) was marginally associated with the standardized operative time. In a multivariate model, tumor circumferential occupation (estimate = 0.03, P = 0.02) and distance to anal verge (estimate = -0.30, P = 0.03) were predictable for the standardized operative time for females (Table 6).

**Table 6:** Determinants for standardized operative time in male and female.

		Male			Female	
Variables		Estimate (SE)	P values	Variable	Estimate (SE)	P values
Univariate						
BMI		0.13 (0.03)	< .0001	Age	0.02 (0.01)	0.05
Temporary diversion		0.46 (0.19)	0.002	Interspinous distance	0.03 (0.01)	0.04
Distance to anal verge		-0.31 (0.08)	0.0001			
Prior abdominal surgery		0.96 (0.31)	0.003			
Pre-chemoradiotherapy		1.06 (0.27)	0.0002			
Interspinous distance		-0.04 (0.01)	0.0002			
Multivariate						
	Intercept	0.35 (0.08)	< .0001	Intercept	1.01 (0.27)	0.02
	BMI	0.10 (0.03)	0.0003	Circumferential	0.03 (0.01)	0.02
Interspinous distance		-0.02 (0.01)	0.001	occupation		
Pre-chemoradiotherapy		0.72 (0.24)	0.004	Distance to anal verge	-0.30 (0.13)	0.03
Prior abdominal surgery		0.79 (0.26)	0.003			

SE: standard error

## Predictability of the model

We then tested predictability of the model using data of 44 patients from other surgeons (Q.L., Y.F. and D.L.). Demographic information of these patients and cancer features were listed in Supporting Information (S1 and S2 Tables). These patients have much similar characteristics as other 155 patients. However, the average of operative time (242.6 ± 57.2 minutes) for these patients were significantly longer than that of patients from surgeon W.C. (166.5 ± 62.8 minutes).

**Fig 2:**
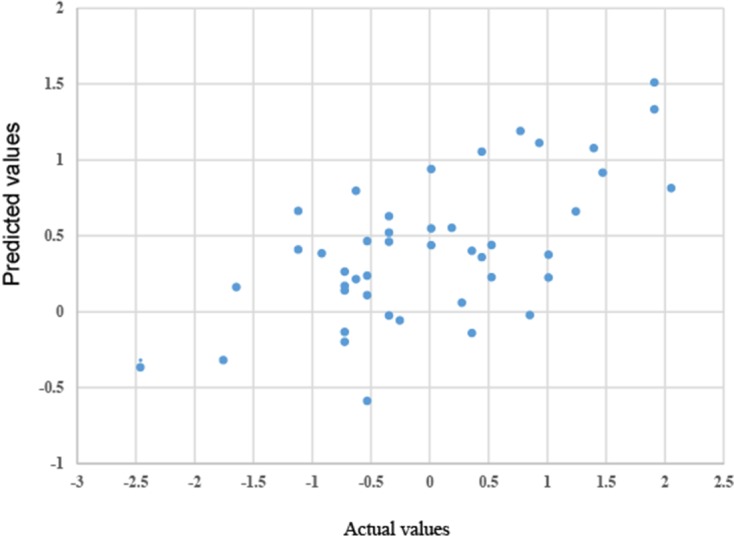
Scatter plot of actual and predicted standardized operative time.

We calculated the standardized operative time for this group of patients by minusing the mean and then dividing the standard deviation. The predicted standardized operative time was calculated based on the built model. The correlation between the two values was analyzed by a linear regression model. The result showed that the slope coefficient was 0.30 with a standard error of 0.06 (P<0.0001), and the estimated correlation r was 0.63 with a 95% confidence interval of 0.41–0.78 (P<0.0001) (Fig 2), suggesting two values were significantly correlated.

## Discussion

This retrospective study includes 199 consecutive patients who had rectal cancer 5–8 cm from the anal verge. All these patients underwent laparoscopic sphincter-sparing low anterior resections with TME and DST. To further control confounding factors to screen only patients’ factors, data from one surgeon were utilized to build models to predict operative difficulty. Our study shows that the main predictors for operative time are: prior abdominal surgery, preoperative chemoradiotherapy, BMI, interspinous distance, and tumor distance to anal verge. Tumor diameter is a predictor for both blood loss and postoperative morbidity. With the well-controlled data, our built model predicts operative difficulty well.

This study utilizes, for the first time, a standardized operative time as the endpoint. This is obtained through a simple transformation method. Operative time is one of the most commonly used endpoints to estimate operative difficulty; however, this is influenced not solely by patient factors, but by many additional factors, such as: different operative procedures, intraoperative complications and the skill level of surgeons. The mean operative time reported in previous studies varies from 153 to over 300 minutes [13, 16, 18, 20]. The absolute value of operative time is not an ideal indicator for operative difficulty, particularly if it is from different studies. The standardized operative time indirectly indicates its percentile ranking of operative difficulty. By using this endpoint, it is possible to pool data from different studies together to increase sample sizes. This will then allow identification of more related predictors and the ability to generate reliable models for predicting operative difficulty applicable for the clinical use. For this purpose, we propose to establish an on line data center to store original data of all published papers.

Preoperative chemoradiotherapy has been shown to reduce local recurrence and improve survival for rectal cancer patients [21, 22]. It considerably reduces tumor size and improves exposure of the surgical field thus helping obtain a safe resection margin [23]. Preoperative chemoradiotherapy has no obvious negative impact on short-term surgical outcomes of laparoscopic resection for rectal cancer [18, 24, 25]. It does however cause tissue edema, fibrosis, extensive mist and exudates which impede the dissection of the tissue, and may increase both the operative time and blood loss during surgery [18]. Similarly, prior abdominal surgery causes formation of adhesions and tissue fibrosis, and conceivably increases the difficulty of laparoscopic resection for rectal cancer [26]. As expected, this study shows that both above preoperative clinical factors are associated with increased operative time.

Laparoscopic surgery for rectal cancer is performed within the pelvic cavity, which limits vision, access, and space. The anatomical parameters, such as prominence of sacral promontory, degree of sacral curves, and size of the pelvis, are associated with operative difficulty. Many complicated pelvic parameters have been applied in previous studies in order to predict their effects on operative difficulty [13, 16, 27–30]; however, not all of these pelvic parameters are related to operative difficulty. Previous studies have shown correlations between longer operative time and a less acutely curved sacrum [30], a smaller pelvic outlet [13], a smaller pelvic diameter [28], and shorter transverse interspinous distance [27]. We have employed two measurements of the pelvis based on CT images, transverse interspinous distance and anteroposterior sacral-pubis distance at the ischial spine level. At the ischial spine, the rectum is approximately 5–8 cm from the anal verge and the pelvis has the narrowest transverse distance. The CT image (Fig 1) clearly displays that the operative field for rectal cancer is strictly limited transversely by the pelvis. In contrast, other major organs, such as the bladder, and the prostate or uterus, can be pushed anteriorly to a certain extent; therefore, the operative field is relatively more flexible in this direction. Our results shows that transverse interspinous distance is associated with operative time [27], but not the sacral-pubis distance. Our study reaffirms that pelvic cavity size is an important factor influencing operative difficulty, and that some critical parameters are more valuable than others.

Our study shows that BMI is a predictor of operative time for men only. The same result was observed in a previous study; however, no detailed data were presented [13]. A previous report shows that visceral fat is more accurate in estimating operative difficulty than regular BMI data [20]. BMI does not consistently reflect body adipose tissue distribution. It has been observed that obese males have more visceral fat, whereas obese females have more subcutaneous fat [31, 32]. This different distribution of fat in males and females may explain that BMI is a predictor only for males. Relatively smaller female sample in our study may also account for the above finding. However, we have found that BMI is an easily obtainable and useful parameter in predicting operative difficulty.

The Akiyoshi group has previously published an excellent study identifying patient’s factors predicting the difficulty of performing laparoscopic low anterior resection for rectal cancer [13]. Compared to their study, our study includes a sample size of twice the participants, as well as having all surgeries performed by the same surgeon, instead of several. It is important to note that there are different definitions of operative times used in both studies. Sphincter preserving resection for rectal cancer 3–4 cm to anal verge has been associated with increased possibility of positive resection margin and a high chance of recurrence [33]. Our study includes patients with rectal cancer 5–8 cm from anal verge, whereas patients with rectal cancer 3–8 cm from anal verge are included in Akiyoshi’s study. Consequently, both studies reveal that BMI, tumor distance to anal verge and pelvic anatomy are predictors for operative difficulty. Tumor depth is a predictor for operative time in their study. Tumor diameter is a predictor for both blood loss and postoperative morbidity in our study. To date, only our study shows that prior abdominal surgery and preoperative chemoradiotherapy are associated with a longer operative time. Our findings suggest that similar results may be obtained using the same laparoscopic procedure in patients having similar features. Increased sample size and control of confounding factors may be helpful in identifying more factors predicting operative difficulty.

One of the major limitations of our study is its retrospective design. The predictability of the model is only examined by the cohort from the same hospital. Only 1/3 of the patients in our study are female, although the incidence of male and female rectal cancer patients is similar in China [34]. Due to the extra costs, only 10.3% of patients were willing to receive preoperative chemoradiotherapy. The average of our patients’ BMI is much lower than that of population in Western Countries, but it is comparable to Chinese colorectal cancer patients reported in a recent study [35]. None of the patients who underwent laparoscopic surgery were converted to open surgery, nor were positive longitudinal resection margins observed in our patients. Only 5 cases had anastomotic leakage. It is not possible to identify determinants for these endpoints, which have been widely used in previous studies. There is no short or long-term follow-up data for these patients. Despite these limitations, this study is valuable because of its sample size, control of confounding factors and having a model with excellent predictability.

## Conclusions

Using this well-defined patient population with a moderate sample size, we identify predictable patients’ factors for operative difficulty. Males and females may have different determinants of operative difficulty. Standardized endpoints will make it possible to significantly increase sample size, by pooling data from different studies, to build more reliable models for clinical use.

## Supporting Information

S1 Table Patients’ demographic and anthropomorphic features, intraoperative and postoperative outcomes. (DOCX)

S2 Table Anatomopathological features of tumors. (DOCX)

## Abbreviations

BMI: body mass index
DST: double stapling technique
TME: total mesorectal excision
TNM: tumor–node–metastasis
UICC: Union for International Cancer Control
