# Supplementary material for: Factors Predicting Difficulty of Laparoscopic Low Anterior Resection for Rectal Cancer with Total Mesorectal Excision and Double Stapling Technique
Source: PLoS One. 2016 Mar 18;11(3):e0151773. doi: 10.1371/journal.pone.0151773 (PMC4798689; doi:10.1371/journal.pone.0151773)
Supplement: S1 Table — (DOCX) [file pone.0151773.s001.docx]

### Supporting Information

**S1 Table. Patients’ demographic and anthropomorphic features, intraoperative and postoperative outcomes.**

|  | Overall | Male | Female |  |
| --- | --- | --- | --- | --- |
|  | 44 | 24(54.5%) | 20(45.5%) | P |
| Age (years) | 57.9 ± 9.9 | 59.0 ± 10.5 | 56.7 ± 9.4 | 0.4496 |
| BMI (kg/m^2^) | 22.3 ± 2.7 | 22.6 ± 2.7 | 22.0 ± 2.7 | 0.4911 |
| Interspinous distance (mm) | 104.9 ± 9.9 | 102.0 ± 10.5 | 108.5 ± 8.1 | 0.0311 |
| Sacrum–Pubis (mm) | 103.2 ± 8.7 | 98.9 ± 8.4 | 108.4 ± 5.9 | 0.0001 |
| Preoperative chemoradiotherapy | 3 (6.8%) | 0 (0%) | 3 (6.8%) | 0.1722 |
| Concurrent diseases | 8 (18.2) | 5 (11.4%) | 3 (6.8%) | 0.6171 |
| Prior abdominal surgery | 3 (6.8%) | 1 (2.3%) | 2 (4.5%) | 0.8695 |
| Operative time (mins) | 242.6 ± 57.2 | 234.8 ± 59.6 | 252.0 ± 54.1 | 0.3260 |
| Blood loss (ml) | 145.0 ± 72.1 | 139.6 ± 80.8 | 151.5 ± 61.5 | 0.5909 |
| Postoperative hospital stay (days) | 12.6 ± 7.6 | 13.2 ± 7.2 | 11.8 ± 8.3 | 0.5516 |
| Temporary diversion | 16 (36.3%) | 6 (22.7%) | 10 (11.6%) | 0.0861 |
| Morbidity | 5 (11.3%) | 3 (6.8%) | 2 (4.5%) | 0.8283 |

Continuous data presented as mean ± standard deviation were analyzed by student t-test, whereas categorical data were examined by Chi-Square test.
