# Supplementary material for: Factors Predicting Difficulty of Laparoscopic Low Anterior Resection for Rectal Cancer with Total Mesorectal Excision and Double Stapling Technique
Source: PLoS One. 2016 Mar 18;11(3):e0151773. doi: 10.1371/journal.pone.0151773 (PMC4798689; doi:10.1371/journal.pone.0151773)
Supplement: S2 Table — (DOCX) [file pone.0151773.s002.docx]

### Supporting Information

**S2 Table. Anatomopathological features of tumors**

|  | Overall | Male | Female | P |
| --- | --- | --- | --- | --- |
| Tumor diameter | 4.7 ± 1.3 | 4.7 ± 1.1 | 4.6 ± 1.5 | 0.8350 |
| Circumferential occupation | 0.6 ± 0.2 | 0.7 ± 0.2 | 0.6 ± 0.3 | 0.2587 |
| Lymph nodes | 17.4 ± 7.3 | 18.0 ± 87.0 | 16.7 ± 7.9 | 0.5555 |
| Stage |  |  |  |  |
| 0 | 1 (2.2%) | 0 (0%) | 1 (5.0%) |  |
| I | 7 (15.9%) | 3 (12.5%) | 4 (20.0%) |  |
| II | 15 (34.1%) | 10 (41.7%) | 5 (25.0%) |  |
| III | 21 (47.8%) | 11 (45.8%) | 10 (50.0%) | 0.9113 |
| T |  |  |  |  |
| 0 | 1 (2.2%) | 0 (0%) | 1 (5.0%) |  |
| 1 | 2 (4.5%) | 0 (0%) | 2 (10.0%) |  |
| 2 | 8 (18.2%) | 3 (12.5%) | 5 (25.0%) |  |
| 3 | 18 (40.9%) | 13 (54.2%) | 5 (25.0%) |  |
| 4 | 15 (34.1%) | 8 (33.3%) | 7 (35.0%) | 0.8260 |
| N |  |  |  |  |
| 0 | 23 (52.2%) | 13 (54.2%) | 10 (50.0%) |  |
| 1 | 16 (36.4%) | 8 (33.3%) | 8 (25.0%) |  |
| 2 | 5 (11.4%) | 3 (12.5%) | 2 (25.0%) | 0.9729 |
| Anal verge (cm) | |  |  |  |
| 5 | 10 (22.7%) | 7 (29.2%) | 3 (15.0%) |  |
| 6 | 11 (25.0%) | 4 (16.7%) | 7 (35.0%) |  |
| 7 | 8 (18.2%) | 3 (12.5%) | 5 (25.0%) |  |
| 8 | 15 (34.1%) | 10 (41.7%) | 5 (25.0%) | 0.5509 |

Continuous data presented as mean ± standard deviation were analyzed by T-test, whereas categorical data were examined by Chi-Square test.
